# Acute-on-Chronic Pancreatitis in a Patient With Ehlers-Danlos Syndrome: A Rare Association

**DOI:** 10.7759/cureus.81189

**Published:** 2025-03-25

**Authors:** Kadi Nguyen, Luisa Ladel, Rebecca Joseph, Seaf Shafique

**Affiliations:** 1 Department of Internal Medicine, Larner College of Medicine, University of Vermont, Burlington, USA; 2 Department of Internal Medicine, Norwalk Hospital, Norwalk, USA; 3 Department of Radiology, Norwalk Hospital, Norwalk, USA

**Keywords:** chronic pancreatitis (cp), connective tissue disease related complications, connective tissue disorder, connective tissue fragility, ehlers danlos syndrome, necrotizing pancreatitis, recurrent acute pancreatitis

## Abstract

Ehlers-Danlos Syndrome (EDS) is a connective tissue disorder known for its vascular complications, but it is rarely associated with chronic pancreatitis (CP). We describe a 32-year-old female with EDS who presented with acute-on-chronic pancreatitis, following prior episodes of pancreatitis and pseudocyst formation two years prior. This patient’s EDS may have predisposed her to recurrent pancreatic inflammation, highlighting the potential role of tissue fragility in pancreatitis susceptibility. This case suggests a need for increased awareness of EDS as a risk factor for pancreatitis and emphasizes the importance of lifestyle counseling to prevent triggers.

## Introduction

Ehlers-Danlos Syndrome (EDS) encompasses a group of connective tissue disorders, characterized by gene alterations causing defective collagen synthesis and processing.

EDS has been linked with acute pancreatitis, though at the time of our review, only one prior case report from Denmark was available in the English literature linking EDS with chronic pancreatitis (CP) [1]. CP typically occurs after multiple episodes of recurrent acute pancreatitis of any cause and, as opposed to acute pancreatitis, is characterized by potentially significant impairment of pancreatic function as well as structural changes, such as atrophy, calcifications, pancreatic duct irregularities, and pseudocysts [2].

EDS is genetically heterogeneous, but many subtypes of EDS have been linked to mutations in a set of genes controlling the cross-linking of collagen, resulting in unstable collagen fibrils. For example, *TNXB*, *AEBP1*, and others have been implicated. EDS occurs at an approximate frequency of 1 in 5,000, with hypermobile EDS (hEDS) being the most common subtype by far [3]. Most types of EDS are inherited in an autosomal dominant fashion. Interestingly, while hEDS follows a dominant inheritance pattern as well, the pathophysiology is not well understood, and underlying genetic, epigenetic, or protein markers remain yet to be identified [4].

EDS is marked by clinical features such as hyperextensive skin, joint mobility, and tissue fragility, which may also affect the vasculature, increasing the risk of organ and vessel rupture [4]. Diagnosis relies on clinical criteria and can be confirmed with genetic testing. There is no curative treatment; management involves regular cardiovascular and ophthalmologic evaluations, as well as physical therapy to preserve joint function.

We report a 32-year-old female with EDS presenting with acute-on-chronic pancreatitis and aim to further explore a possible pathophysiologic connection between EDS, the development of pancreatitis, and progression from acute to chronic pancreatitis.

## Case presentation

A 32-year-old female from the United States with EDS and a history of gallstone pancreatitis complicated by necrotizing pancreatitis and pseudocyst formation presented with acute left upper quadrant abdominal and back pain. She had been hospitalized two years prior for gallstone pancreatitis and underwent endoscopic retrograde cholangiopancreatography (ERCP) with bile duct stone extraction and cholecystectomy. Over the following month, she was readmitted for large pancreatic pseudocysts and necrosis, requiring multiple endoscopic ultrasound (EUS)-guided cyst gastrostomies, pseudocyst drainage, and direct endoscopic necrosectomy. She reported similar symptoms with her current presentation.

**Figure 1:**
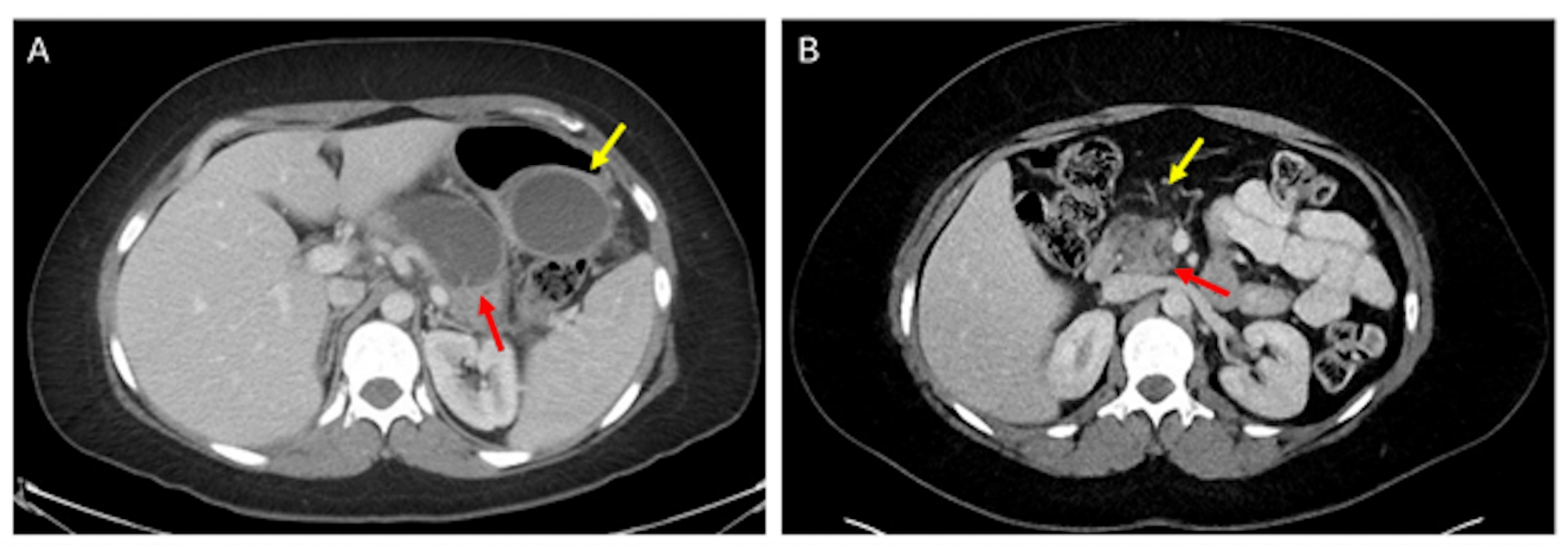
CT images of pancreatic cyst evolution Axial CT scan of the abdomen with IV contrast obtained during the patient's first episode of pancreatitis (A) demonstrates fluid-attenuating lesions within the body of the pancreas (red arrow) and along the gastric wall (yellow arrow), which both represent pseudocysts in the setting of recent pancreatitis and demonstrate the patient's complicated course. Axial CT scan of the abdomen with IV contrast from two years later (B) demonstrates pancreatic head edema (red arrow) and surrounding fat stranding (yellow arrow), consistent with a repeat episode of acute pancreatitis. CT: computed tomography

On admission, the patient was afebrile and hemodynamically stable. Labs revealed leukocytosis with neutrophilic predominance and a rise in C-reactive protein (from 16.6 g/dL to 40.8 g/dL) on admission, indicating a new inflammatory or infectious process. Liver function tests were fairly unremarkable, making biliary obstruction unlikely. Lipase was normal. Triglycerides were normal as well, ruling out hypertriglyceridemia as a possible etiology of acute pancreatitis (Table 1). Abdominal computed tomography (CT) imaging revealed edema surrounding the pancreatic head with mild fat stranding, pancreatic atrophy, and two fluid-attenuated lesions (2.7-2.9 cm) in the pancreatic head and body (Figure 1). Without interval imaging, it was unclear whether these lesions were new or a remnant of prior necrotizing pancreatitis.

**Table 1:** Laboratory values obtained upon admission

Laboratory test	Value (unit)	Reference range (unit)
White blood cell count	11.8 x 10^9^/L	3.5-10.0 x 10^9^/L
C-reactive protein (CRP)	40.8 mg/L	0.0-4.9 mg/L
Total bilirubin	1.1 mg/dL	0.0-1.2 mg/dL
Alkaline phosphatase	82 U/L	30-146 U/L
Alanine transaminase (ALT)	70 U/L	10-55 U/L
Aspartate transaminase (AST)	15 U/L	10-50 U/L
Lipase	23 U/L	13-60 U/L
Triglycerides	73 mg/dL	0-149 mg/dL

Despite a normal lipase level, the patient met diagnostic criteria for acute pancreatitis based on her characteristic pain presentation and classic imaging findings, including pancreatic head edema and surrounding fat stranding (Figure 1). The pancreatic atrophy also suggested a degree of CP, leading to a diagnosis of acute-on-chronic pancreatitis. Treatment included supportive care with high-volume intravenous fluids, pain management, and gradual dietary reintroduction.

**Figure 2:**
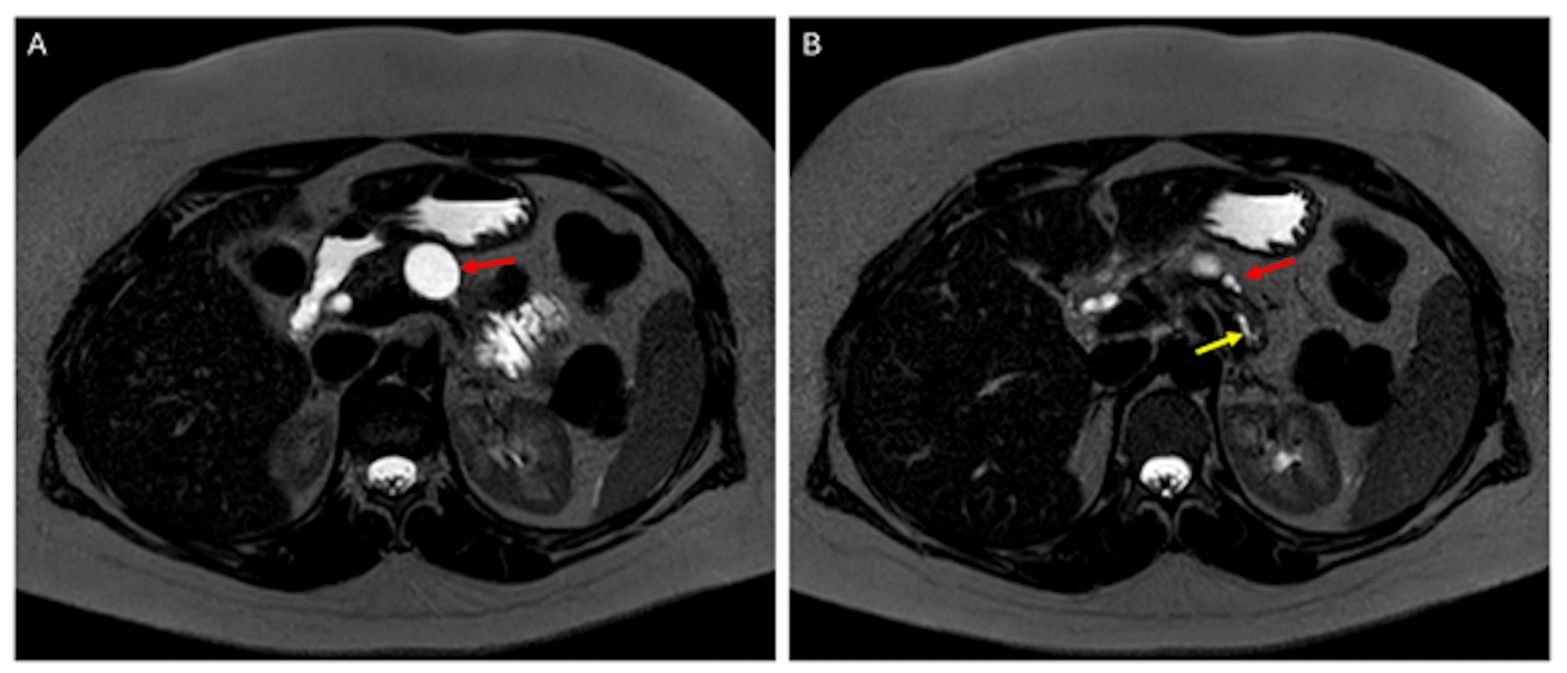
MRI images of remaining pancreatic cysts after resolution of pancreatitis Axial thin MRCP image of the abdomen (A) demonstrates a dominant, T2-hyperintense cyst within the pancreatic body (red arrow). One slice inferior (B), there are two adjacent smaller pancreatic cysts (red arrow) and dilation of the main pancreatic duct within the partially atrophic pancreatic tail (yellow arrow). These findings are consistent with local complications from acute pancreatitis and showcase the development of chronic pancreatitis. MRI: magnetic resonance imaging; MRCP: magnetic resonance cholangiopancreatography

**Figure 3:**
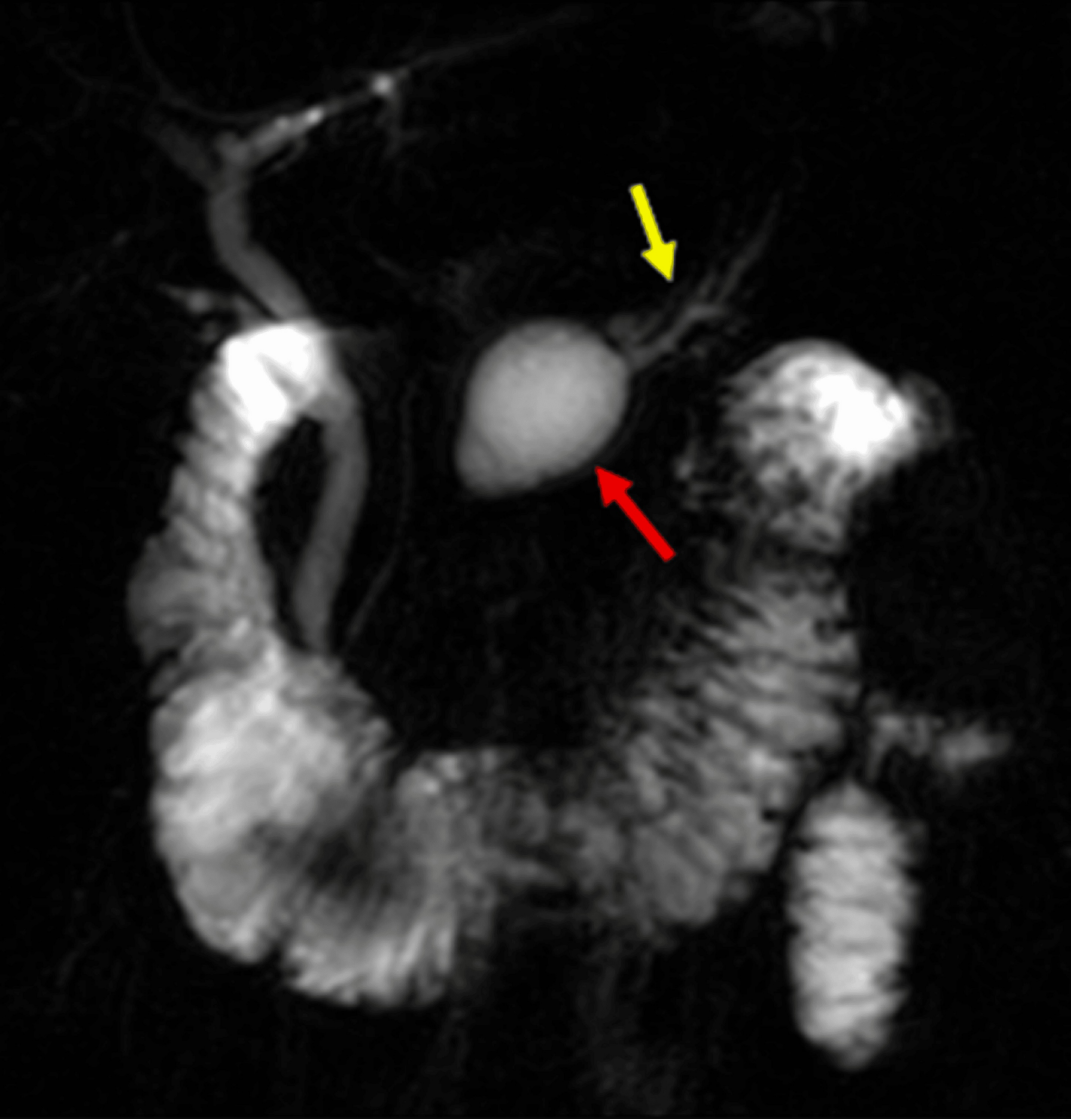
MRCP image of dominant pancreatic cyst Coronal MRCP MIP sequence shows the dominant pancreatic body cyst (red arrow) and dilation of the distal main pancreatic duct (yellow arrow); both are findings characteristic of pancreatitis. MRCP: magnetic resonance cholangiopancreatography; MIP: maximum intensity projection

During her hospitalization, the patient advanced to a low-fat diet and achieved pain control through tapering hydromorphone and oxycodone. She was counseled on low-fat diet adherence and alcohol avoidance, then discharged with a referral to a pancreatitis specialist. On follow-up, magnetic resonance cholangiopancreatography (MRCP) imaging showed remaining cysts consistent with CP (3.3 cm and 0.8 cm; Figures 2-3). However, in the setting of a pancreatitis-friendly diet, the patient reportedly noted improvement in symptoms and pain control.

## Discussion

CP is defined as prolonged inflammation of the pancreas, causing irreversible morphological changes and some loss of function [5]. CP is typically associated with gallstones, alcohol, or drug use, accounting for approximately 70% of cases [6]. However, 20% of cases are idiopathic. Several studies have reported EDS-related vascular issues (splenic rupture [7] and hepatic arterial aneurysm [8]) and gastrointestinal disorders, including hiatal hernias and bowel perforations [9]. To our knowledge, there is only one other case study on EDS and CP in the current literature [1].

Two years prior to this hospitalization, our patient was evaluated for skin hyperelasticity, joint mobility, and arthralgia and was diagnosed with EDS by a rheumatologist six months after her initial pancreatitis episode. For the two years between pancreatitis episodes, the patient had been asymptomatic from a CP standpoint, without abdominal pain, development of pancreatogenic (type 3c) diabetes, or evidence of exocrine pancreatic dysfunction.

During the present hospitalization, our patient’s presentation of classic pancreatitis pain and imaging findings of pancreatic atrophy suggested CP, likely from prior necrotizing pancreatitis. Her lipase level was within normal limits; however, this does not preclude the diagnosis of acute-on-chronic pancreatitis, as the patient met the criteria for characteristic pain and imaging findings. In fact, while lipase elevation is a typical finding in patients with acute pancreatitis, it has been observed that patients with chronic or acute-on-chronic pancreatitis often present without significant elevation in lipase and/or amylase levels, due to pancreatic atrophy and progressive destruction of functional pancreatic tissue that would be able to release such enzymes into the bloodstream upon injury [10,11].

Despite extensive workup, no definitive trigger for the patient’s acute-on-chronic pancreatitis emerged. Following initial gallstone pancreatitis and cholecystectomy, imaging showed no choledocholithiasis or obstructive factors; triglycerides and calcium were normal, and she was not on pancreatitis-associated medications. Additionally, there was no indication of autoimmune pancreatitis or inflammatory bowel disease. She reported no prior or current tobacco consumption and endorsed only minimal alcohol use (two drinks or less per week), though some studies suggest alcohol-related CP requires more heavy alcohol use, including episodes of binge drinking [12], or heavy alcohol use of five or more drinks daily for at least five years [13]. However, persistent alcohol use after the development of CP is known to increase the risk of recurrent pancreatitis [13], and although in our patient it was minimal, it may have contributed to this acute exacerbation.

Similarly, our patient had a very complicated course during her first episode of acute pancreatitis several years prior to this current presentation, where she developed necrotizing pancreatitis and large pseudocysts requiring multiple interventions, ultimately leading to CP (Figure 1). While about 20% of acute pancreatitis cases may be associated with complications such as these, our patient's aggressive course, despite timely treatment of biliary obstruction and subsequent recurrent episodes of acute-on-chronic pancreatitis without an identifiable trigger, raises the question of an intrinsically higher risk for pancreatitis in EDS patients, possibly associated with EDS-related connective tissue fragility.

While we did not observe any differences in the symptomatology, we found this potential propensity towards the development of chronic or recurrent pancreatitis, without classic offending triggers, to be the most striking difference in presentation between EDS-related and non-EDS-related pancreatitis patients. This supports our hypothesis that some of the CP cases considered idiopathic at this time may be related to underlying EDS or potentially even other connective tissue diseases.

CP is often linked to toxic-metabolic factors, but genetic pathologies can also play a role. Genetic risk factors include mutations in *PRSS1*, *CFTR*, *SPINK1*, and chymotrypsin C, which serve as modifier genes increasing susceptibility to CP [14,15]. These variants are more common in patients of European and Asian ancestry, and their contribution to pancreatitis severity remains under investigation. Unfortunately, we were unable to perform genetic testing on our patient.

Though limited, evidence suggests a link between EDS and pancreatitis via pancreatic capillary vessel leaks as the main driver of pancreatic damage and inflammation [1]. It is believed that EDS-associated tissue fragility may contribute to capillary damage, creating a chronic inflammatory state and the potential for chronic atrophy and fibrosis in the pancreas [16-18]. This damage could explain our patient's pancreatic fibrosis.

In the other case study known to us that links EDS and CP, the patient’s demographics and presentation closely mirrored our patient’s: female sex and similar age, presentation for multiple episodes of severe abdominal pain over several years, no alcohol or drug use disorder, no identifiable trigger medications, normal calcium and triglyceride levels, and imaging findings of an atrophic and fibrotic pancreas with ductal dilation (Figure 3). Neither patient showed evidence of obstructing gallstones [1]. Interestingly, lipase levels were normal in this patient's case as well, although she presented with an elevated serum amylase of 486 U/L during one of her pancreatitis episodes. Unlike our patient, the other patient also endorsed a significant weight loss of 5 kg over six weeks. Notably, both patients experienced recurrent admissions for abdominal pain consistent with acute-on-chronic pancreatitis, and symptoms seemed to arise without identifiable classic triggers of pancreatitis; however, imaging findings proved progressive CP. The other patient, unlike our patient, underwent genetic testing for common gene mutations with known associations with pancreatitis, including cystic fibrosis testing; however, she tested negative for all. It is, however, interesting to note that both parents in that case had a history of CP as well, although, in the case report, the authors argue that an association with alcoholic pancreatitis was likely in both parents. Nonetheless, it would be reasonable to assume that persons with a genetic predisposition to pancreatitis development would also be more susceptible to the development of alcohol-related pancreatitis with excessive alcohol consumption.

In summary, we hypothesize that vascular collapse, due to connective tissue insufficiency in EDS, may increase the risk of developing CP through a persistent and recurrent inflammatory state within the pancreas, which may also be a driver for recurrent acute-on-chronic pancreatitis attacks without otherwise identifiable triggers.

## Conclusions

In summary, our case describes a young female patient with EDS and acute-on-chronic pancreatitis. Although the patient's first episode of acute pancreatitis was deemed to be secondary to obstruction from a gallstone, she had a very complicated course with the development of pseudocysts and CP, despite timely relief of the obstruction. In addition, she subsequently experienced episodes of acute-on-chronic pancreatitis without identification of a known trigger, overall raising the question of whether EDS may have contributed as a predisposing factor to the development of CP, as well as recurrent episodes of acute pancreatitis, with a hypothesis of connective tissue fragility and capillary vessel damage driving such a pathophysiologic process. Of course, given the limitations of our report and the lack of genetic or histopathological confirmation of such pancreatic tissue fragility in the literature so far, this is difficult to ascertain, especially considering the lack of readily available large-scale epidemiological data on this topic. Further investigation is therefore required to fully understand any potential connection between EDS and CP, with genetic and epigenetic studies presenting particularly interesting avenues for future studies.

Nonetheless, clinicians should be mindful of a potential association between EDS and both acute and CP, given the multitude of factors that could induce potentially devastating sequelae, including lifelong complications with persistent abdominal pain and irreversible pancreatic damage. Patients with EDS should be counseled on this possible link and advised to avoid known triggers of pancreatitis, such as alcohol consumption, smoking, and untreated dyslipidemia or hypertriglyceridemia, as they may harbor an intrinsic predisposition for the development of pancreatitis.
